# Supplementary material for: Chromothripsis during telomere crisis is independent of NHEJ, and consistent with a replicative origin
Source: Genome Res. 2019 May;29(5):737–49. doi: 10.1101/gr.240705.118 (PMC6499312; doi:10.1101/gr.240705.118)
Supplement: Supplemental Material [file supp_gr.240705.118_Supplemental_file_1.zip › contigs/annotated_contigs/DB114/contig.3.DB114_length_640_mean_cov_6.796875.docx]

**DB114_length_640_mean_cov_6.796875**

```
AAAAAAGAAAAAAAAAATACAAAAGATAATAATATAAAAGATTCCAAAA|AAAACCAAAAAACAAAGACAGAAAGAAAGAAAGGAAAAA
                                                  >chr14:27679260-27679623 - E=1e-200 p=
TTTGAGAAAAACAGCCACTACCTATTGAGAAAAGGAAAAAAAAAAAACACACACACACACAAATAAAATCTACCATGAATACATAAGAG
1e-02
AACAGACAGCAACCATGAAACAAGAATGAGATTCCTTGTCAAAGAAATATTCAAAAGTTTTAAAAGGTATTTTTATACATTAACATTTT

GATGATATGGCTGAAAACTCAGTAAGTGTTGAAAGAAAATGCTAAGGATATTTCCCAGACATTATGGCAAAGAAACAAGCAGATGAATA

GTAAATGAGGAAAGAGCAAAAAATTAGATGTCCTTATTATAAGGCCCTACATCC|A|TATATATATATATTAATATATATATATATTAA
                                                       >chr2:170324426-170324478 + E=1e
TATTAATTATATATATATATA|T|ATTAATGTTTTTGTAGCAGAATCTTACTAAGTAGGTTCCAAAGTTTCTAAAAGTTTCTCCTTTGA
-02                    >chr14:27678341-27678517 - E=4e-94
ATGCATTTCAAACACTATTTAGACAGTGTGGCAAAATATAGAACGGACTAATGGAAGCAAAGAAAGTTGATTGGATTACTAAATTGATT

TTAGAAAGTGGATAATAAGACA
```
